# Endoscopic submucosal dissection for a rectal tumor behind a rectal fold with novel two-step utilization of a traction device

**DOI:** 10.1055/a-2436-1178

**Published:** 2024-10-25

**Authors:** Yoshiaki Ando, Shunsuke Yoshii, Tomoki Michida, Ryu Ishihara

**Affiliations:** 1 53312 Gastrointestinal Oncology, Osaka International Cancer Institute, Osaka, Japan

Colorectal endoscopic submucosal dissection (ESD) can be challenging depending on the location of the lesion 1 2 . A tangential endoscopic approach is key to success. Herein, we describe successfully performing ESD for a rectal cancer using a novel means of achieving a tangential endoscopic approach ( Video 1 ).

Endoscopic submucosal dissection (ESD) for a rectal tumor behind a rectal fold with novel two-step utilization of a multiloop traction device for lifting the adjacent oral-side mucosa and then for suturing the post-ESD ulcer.Video 1

A 63-year-old man was referred to our hospital for endoscopic treatment of a rectal neoplasm. Colonoscopy revealed a 20-mm laterally spreading tumor behind the upper valve of Houston ( Fig. 1 ). The transverse rectal fold in the forward view made it difficult to approach the oral side of the lesion ( Fig. 2 **a** ) and a retroflex maneuver enabled only a perpendicular approach. We therefore used a multiloop traction device (MLTD) (Boston Scientific, Tokyo, Japan) 3 , not to apply traction to the lesion, but to lift the rectal wall on the oral side of the lesion ( Fig. 2 **b** ), markedly improving accessibility to that side of the lesion in the forward view. En bloc resection by ESD was then achieved under stable tangential endoscopic view. The MLTD was then used to complete closure of the post-ESD ulcer. The attached oral-side loop of the MLTD ( Fig. 2 **b** ) was removed and traction applied to its middle loop ( Fig. 2 **b** , Fig. 3 **a** ), enabling fixation to the anal side of the post-ESD ulcer ( Fig. 3 **b** ). This made the post-ESD ulcer narrower, facilitating smooth closure with endoclips. The patient was discharged 4 days after ESD having had no adverse events. Histopathological examination showed a well-differentiated intramucosal adenocarcinoma with negative resection margins.

**Fig. 1:**
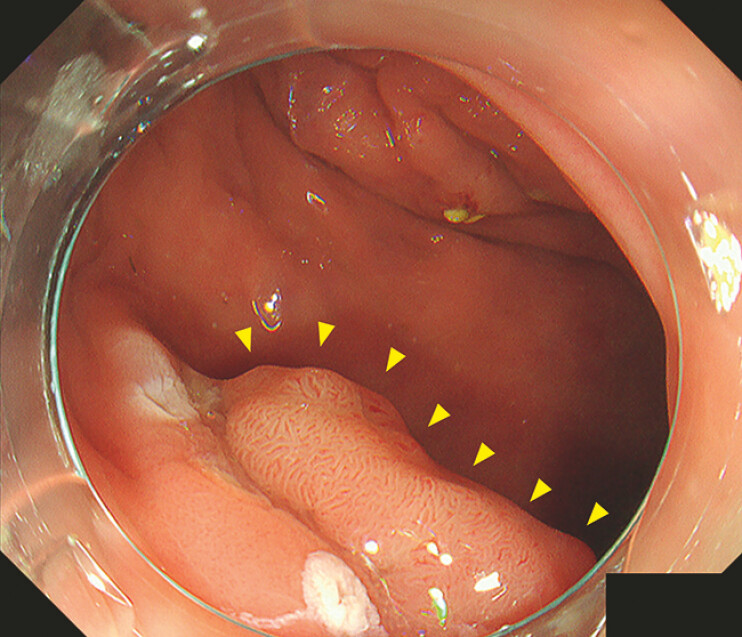
Endoscopic view of a 20-mm laterally spreading tumor located behind the upper valve of Houston.

**Fig. 2:**
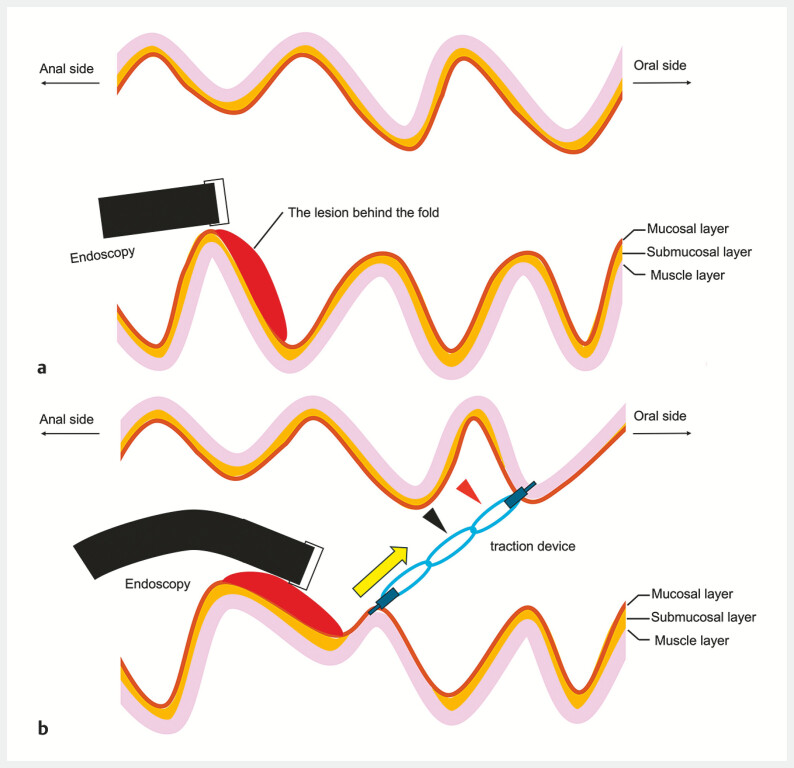
The endoscopic approach to the oral side of a lesion located behind a rectal fold. **a** The lesion was located behind the fold. **b** The multiloop traction device (MLTD) was used to lift the rectal wall on the oral side of the lesion (yellow arrow), to enable endoscopic submucosal dissection (ESD) of the lesion. For closure of the post-ESD ulcer, the attached oral-side loop (red arrowhead) was removed and traction applied to the middle loop (black arrowhead) (see Fig. 3 **a** ).

**Fig. 3:**
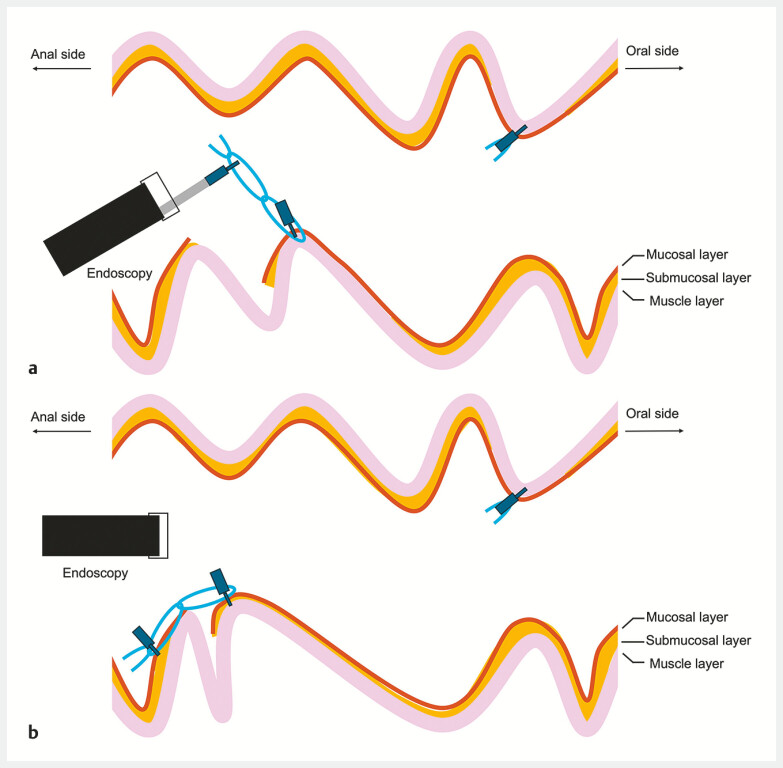
Clip closure of the post-ESD ulcer using the traction device. **a** After removal of the oral-side loop, traction toward the anal side was applied to the middle loop. **b** The middle loop could then be fixed to the anal side of the post-ESD ulcer, which had become narrower.

Location of a tumor behind a fold can pose difficulties during colorectal ESD. Our novel usage of a traction device is an effective means of improving the endoscopic approach and is doubly useful in that it facilitates clip closure after ESD.

Endoscopy_UCTN_Code_TTT_1AQ_2AD_3AD

## Notes

Endoscopy E-Videos https://eref.thieme.de/e-videos
*E-Videos* is an open access online section of the journal *Endoscopy* , reporting on interesting cases and new techniques in gastroenterological endoscopy. All papers include a high-quality video and are published with a Creative Commons CC-BY license. Endoscopy E-Videos qualify for HINARI discounts and waivers and eligibility is automatically checked during the submission process. We grant 100% waivers to articles whose corresponding authors are based in Group A countries and 50% waivers to those who are based in Group B countries as classified by Research4Life (see: https://www.research4life.org/access/eligibility/ ). This section has its own submission website at https://mc.manuscriptcentral.com/e-videos .
